# Fluid Volumes Longitudinal Modeling to Predict Atrophy and Fibrosis in Neovascular Age-Related Macular Degeneration

**DOI:** 10.1016/j.xops.2026.101190

**Published:** 2026-04-15

**Authors:** Stefano Mercuri, Stefan Sacu, Sophie Frank-Publig, Johannes Schrittwieser, Markus Gumpinger, Oliver Leingang, Ursula Schmidt-Erfurth, Usha Chakravarthy, Hrvoje Bogunovic, Gianni Virgili, Gregor S. Reiter

**Affiliations:** 1 Department of Ophthalmology and Optometry, Medical University of Vienna, Vienna, Austria; 2 Department of Neurosciences, Psychology, Drug Research, and Child Health, Eye Clinic, University of Florence, AOU Careggi, Florence, Italy; 3 RetInSight GmbH, Vienna, Austria; 4 Center for Experimental Medicine, Institute of Clinical Science, Queen’s University Belfast, Belfast, UK; 5 Center for Medical Data Science (Institute of Artificial Intelligence), Medical University of Vienna, Vienna, Austria; 6 IRCCS – Fondazione Bietti, Rome, Italy

**Keywords:** Atrophy, Fibrosis, Fluid volumes, Macular neovascularization, Subretinal hyperreflective material

## Abstract

**Purpose:**

To evaluate the impact of retinal fluid volumes on the development of atrophy and fibrosis in neovascular age-related macular degeneration (nAMD) during routine care.

**Design:**

Retrospective longitudinal study.

**Participants:**

Treatment-naïve eyes with nAMD from the Vienna Imaging Biomarker Eye Study (2007–2018), initiating anti-VEGF therapy.

**Methods:**

Volumes of intraretinal fluid (IRF), subretinal fluid (SRF), and pigment epithelial detachment (PED) were automatically quantified on OCT using an approved artificial intelligence algorithm within the 1-, 3-, and 6-mm ETDRS regions. Macular neovascularization (MNV) type and baseline presence of subretinal hyperreflective material (SHRM) were assessed. Fluid volumes were modeled as time-dependent biomarkers using repeated-measure Cox models to evaluate cumulative effects over time on the development of atrophy and fibrosis, with hazard ratios calculated for quartiles (Qs) at each visit. Associations with change in visual acuity (VA) were performed in a subset of eyes with ≥12 months of follow-up.

**Main Outcome Measures:**

Retinal fluid volumes, development of atrophy and fibrosis, and change in VA.

**Results:**

A total of 1060 eyes of 998 patients were included. Higher IRF volumes increased the risk of both atrophy and fibrosis (all *P* < 0.001). Subretinal fluid reduced the risk of atrophy (Q4, *P* ≤ 0.001) and fibrosis (1-mm, *P* = 0.028) in the 1- and 3-mm regions, but increased the risk of fibrosis in the 6-mm region (*P* ≤ 0.031). Higher PED volumes increased the risk of atrophy in the 6-mm region (*P* < 0.001), and fibrosis at all locations (*P* ≤ 0.002). Age and baseline presence of SHRM were associated with atrophy (*P* < 0.001), whereas type 2 and mixed/undefined MNVs were associated with both atrophy (*P* < 0.001) and fibrosis (*P* ≤0.004). In 552 eyes, time-related presence of fibrosis was associated with VA decline at all locations (*P* < 0.001). In all areas, Q4 of IRF was associated with linear VA decline (*P* <0.001). Larger SRF volumes (Q4) in the 1-mm region were associated with better vision (*P* <0.05). Higher PED volumes (Q3, Q4) were linked to VA decline at all locations (*P* < 0.001).

**Conclusions:**

Assessment of the cumulative effects of retinal fluid volumes during anti-VEGF therapy using time-dependent biomarker modeling revealed notable associations with risk of atrophy, fibrosis, and VA decline.

**Financial Disclosure(s):**

Proprietary or commercial disclosure may be found in the Footnotes and Disclosures at the end of this article.

Age-related macular degeneration is a degenerative retinal disease, which in its end stage leads to visual loss, carrying a high socioeconomic burden for patients and health care systems worldwide.^1^, ^2^, ^3^, ^4^ The onset of macular neovascularization (MNV), denominated neovascular age-related macular degeneration (nAMD), leads to a rapid decrease in visual acuity (VA) if the disease is suboptimally treated or if treatment is delayed. Appropriate and timely treatment with anti-VEGF agents tailored to the patient’s needs has been shown to preserve vision.^5^^,^^6^ Various biologics that inhibit VEGF have been developed with the aim of increasing the response rate to treatment, improving the duration of the effect to extend the retreatment intervals, and have been tested in major randomized controlled trials.^7^^,^^8^

Despite the efficacy of these treatments, long-term assessments reveal that initial gains in function are reduced because of the development of end-stage disease complications, mainly macular fibrosis and macular atrophy.^9^, ^10^, ^11^ Notably, the visual and anatomic outcomes in the routine clinical practice setting compared with randomized controlled trials are uniformly worse, probably arising from the disparities in the populations studied, because randomized controlled trials have strict inclusion and exclusion criteria with structured follow-up and retreatment schedules. By contrast, in routine clinical practice settings, there may be suboptimal patient compliance arising from the economic burden of these treatments to patients’ families and health care bottlenecks, as well as loss at follow-up.^12^, ^13^, ^14^ Therefore, a knowledge of prognostic biomarkers that can predict the occurrence and timing of macular fibrosis and macular atrophy may be helpful to shape treatment strategies and patient counseling. Fibrosis is a common outcome in nAMD and one of the main reasons for patient discharge and treatment discontinuation because it is associated with poor VA and irreversible visual loss.^15^^,^^16^ Prevalence of macular fibrosis is as high as 32% and 56% at 1- and 5-year follow-up, respectively.^17^ Moreover, the presence of subretinal hyperreflective material (SHRM) and type 2 MNV has been previously associated with the development of macular fibrosis.^18^

Several studies have noted that macular atrophy may be present in 10% to 40% of eyes before the development of MNV. Other studies indicate an incident rate of between 10% and 50% at 2-year follow-up and 80% at 8 years.^19^, ^20^, ^21^, ^22^, ^23^

The impact of fluid on vision has been extensively studied because the presence of intraretinal fluid (IRF) at baseline and along follow-up has been strongly associated with poorer visual outcomes and subretinal fluid (SRF) has shown a milder but still relevant impact on vision than IRF.^24^, ^25^, ^26^, ^27^, ^28^

Prevention of end-stage complications related to nAMD represents an important goal of treatment, and efforts should be made to determine the morphologic biomarkers carrying prognostic significance, which would change the treatment approach, from the treatment regimen to the choice of anti-VEGF agent. Artificial intelligence (AI) algorithms have been validated to accurately identify and quantify fluid volumes on spectral-domain OCT (SD-OCT) within the macula in nAMD, providing indicators of MNV activity during follow-up.^26^, ^27^, ^28^, ^29^, ^30^, ^31^ Objectively quantifying these findings allows for precise patient monitoring and tracing of fluid dynamics within the patient’s macula. Indeed, the value of longitudinal biomarker modeling in risk prediction has been increasingly acknowledged.^32^, ^33^, ^34^

Therefore, the aim of our study was to combine information on the dynamics of retinal fluid volumes with MNV type and SHRM as predictors for development of macular fibrosis and macular atrophy, thus providing insight into potential independent prognostic factors for these severe, visually disabling end-stages of nAMD.

## Methods

### Study Patients

All data belonged to the Vienna Imaging Biomarker Eye Study registry. All records belonged to databases of the Macula Department, Department of Ophthalmology, Medical University of Vienna, Austria, with dates ranging from 2007 to 2018. Databases were filtered for treatment-naïve nAMD with a baseline SD-OCT and ≥1 follow-up, and 1127 eyes of 1049 patients were included, as previously published.^27^

The Ethics Committee of the Medical University of Vienna approved both the retrospective registry and this retrospective analysis using the registry data, and informed consent was waived for this retrospective analysis. This study conformed to the Declaration of Helsinki and the principles of Good Scientific Practice.

### Main Outcome Measures

Spectral-domain OCT volumes at each follow-up were obtained using CIRRUS HD-OCT III (Carl Zeiss Meditec, Inc) or Spectralis HRA + OCT (Heidelberg Engineering).

OCT volumes of 1127 eyes were segmented with a validated, commercially available, automated deep-learning fluid segmentation algorithm (Fluid Monitor, Version 2.5, RetInSight) for the volume of IRF, SRF, and pigment epithelial detachment (PED), as described in previous studies.^35^^,^^36^ Fluid was analyzed in the 1-, 3-, and 6-mm disc area centered on the fovea, following the standard ETDRS macular grid.

Qualitative variables from SD-OCT volumes were graded by 2 independent graders (S.M. and S.F.). To avoid disagreement, a third grader (G.S.R.) provided the final assessment. At baseline, based on SD-OCT volumes data, MNV type could be defined as “type 1,” “type 2,” “type 3,” and “mixed”; if the MNV type was not clearly identifiable, it was graded as “undefined.” Because of the small number of cases in the subcohorts, mixed and undefined MNVs were grouped into a new category called “mixed/undefined.” Subretinal hyperreflective material at baseline was classified as “present” or “absent.”^37^ Subretinal HRM was defined as HRM in the subretinal space, with a distinguishable and clearly identifiable retinal pigment epithelium (RPE) layer beneath the material. Subretinal HRM was only graded at baseline because this lesion component either resolves during treatment or converts to fibrosis in an angiofibrotic switch.^38^

At each follow-up, the presence of macular fibrosis or macular atrophy was assessed based on SD-OCT data from the entire area of retina covered by volume scans. Based on SD-OCT data alone, fibrosis was defined as the presence of HRM at the level of the outer retina with an undistinguishable underlying RPE band.^16^ Atrophy was defined as areas of complete RPE and outer retinal atrophy with >250 μm in size, according to Classification of Atrophy Meetings group classification.^39^

Although the scans were being assessed, 67 eyes (5.9%) were excluded because of poor image quality (impossible to identify retinal layers, distinguish their integrity, or identify the fovea); the remaining eyes were included in the base data set for further analysis. Scans were assessed masked.

Visual acuity measures (logarithm of the minimum angle of resolution [LogMAR]) were also collected when available.

### Statistical Analysis

To overcome the skewed nature of the data arising from a proportion with nil volumes, we computed the integral of fluid volumes during follow-up, meaning that the cumulative amount of each fluid type over time was a time-dependent exposure. Areas under the curve (AUCs) of each fluid compartment were computed at the individual level, using iterative commands for each eye at each month of follow-up, with codes and explanations on their use available in Appendix S1 (available at www.ophthalmologyscience.org). We used quartiles (Qs) of each fluid type at each follow-up point to model the exposure as a nonparametric covariate, with Q1 representing the absence of (or minimal) fluid and Q4 the highest fluid volume within the fluid compartment.

We used repeated-measure survival analysis to model the time-dependent effect of IRF, SRF, and PED on the development of atrophy and fibrosis. The changing effect of fluid over time was modeled as Qs of fluid at each follow-up for those eyes with data present. Cox models accounted for repeated measures using a robust variance estimator clustering on each eye. This approach addresses potential model misspecification, using a “sandwich-type” covariance matrix estimator that remains consistent even when the model's assumptions (such as proportional hazards) do not strictly hold, allowing for valid statistical inferences about covariate effects.^40^

A test for linear trend of hazard ratios (HRs) over Qs of fluid was obtained using Qs as a numerical variable (1–4).

Mixed models were used to present fluid compartments over time. A Bonferroni correction was applied to each primary analysis set, including 27 comparisons of Qs 2, 3, and 4 versus 1 across 1-, 3-, and 6-mm discs of the EDTRS grid; thus, a *P* value of 0.0019 was the threshold for statistical significance. In addition, the Benjamini-Hochberg false discovery rate multiple comparison was performed to generate adjusted *P* values.

Methodology of main analyses is represented in Appendix S1.

Model fit based on the same data was compared using Akaike information criteria and Bayesian information criteria (BIC), with a difference of 5 indicating a better fit of the model with a lower score.

Linear mixed models were used to investigate the effect of fluid volumes and the presence of atrophy/fibrosis on the change in VA over follow-up. To limit the number of covariates in each model, the simultaneous effect of the fluid compartments (IRF, SRF, and PED) was investigated in different models by area (1, 3, and 6 mm). The lowest Q (Q1) was used as the reference. Follow-up time, as a linear term, was included both as a covariate in the fixed part of the model and as a random slope; an interaction term was used between follow-up time and atrophy/fibrosis to explore their progressive effects on VA. All the analyses, including VA measures, were carried out on a subset of patients with a recorded VA follow-up of ≥12 months.

## Results

The analysis included 1060 eyes from 998 patients (62 bilateral patients at baseline). After the exclusion of 94 eyes with both atrophy and fibrosis at baseline, 966 eyes from 912 patients (54 bilateral patients at baseline) were included in the analyses of patients who developed atrophy (429 eyes, 44.4%) or fibrosis (275 eyes, 28.5%) during the entire follow-up.

At baseline, the median age was 79 (interquartile range, 72–84) years, and the median follow-up was 24 months (interquartile range, 12–48 months). Prevalence of MNV types was as follows: 516 eyes (53.4%) with type 1 MNV, 177 eyes (18.3%) with type 2 MNV, 186 eyes (19.3%) with type 3 MNV, and 87 eyes (9.0%) with mixed/undefined MNV.

Confidence interval of each fluid compartment volume at each timepoint and baseline Qs cutoff for each fluid compartment are depicted in Figure S1 and Table S1 (available at www.ophthalmologyscience.org). All compartments had an initial decrease, with IRF showing a gradual increase thereafter.

### Development of Atrophy

These analyses included 719 eyes with no atrophy at baseline, with 291 developing atrophy during follow-up. The survival analysis estimated proportion free of atrophy at 1 to 5 years was 81%, 67%, 53%, 42%, and 27%, respectively.

**Figure 2:**
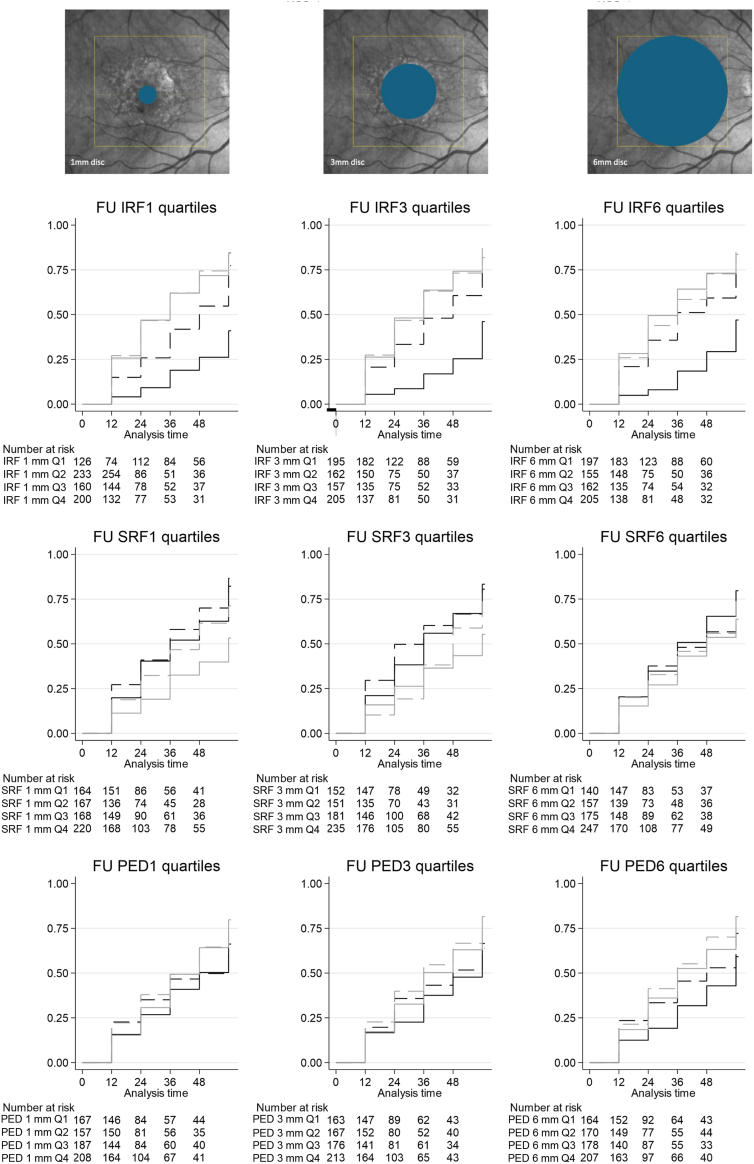
Risk of atrophy along follow-up (months) for different fluid quartiles (Qs): intraretinal fluid (IRF), subretinal fluid (SRF), and pigment epithelial detachment (PED) fluid volumes at 1 mm (left column) show an increased risk of atrophy for higher Qs of IRF and PED volumes, with a protective effect toward atrophy for Q4 of SRF. Intraretinal fluid, SRF, and PED fluid volumes at 3 mm (middle column) show an increased risk of atrophy for higher Qs of IRF, with a protective effect for Q3 and Q4 of SRF. Intraretinal fluid, SRF, and PED fluid volumes at 6 mm (right column) show an increased risk of atrophy for higher Qs of IRF and PED compared with Q1. Line patterns are follows: Q1, solid black; Q2, dashed black; Q3, dashed gray; and Q4, solid gray. FU = follow-up

Figure 2 and Table 2 show the HRs and incidence of atrophy development for fluid compartment Qs at each of 3 ETDRS subfields.

**Table 2:** Fluid Volumes and Risk of Atrophy

Atrophy			
Volume Quartile	1 mm disc Hazard ratio (95% CI); *P* value	3 mm disc Hazard ratio (95% CI); *P* value	6 mm disc Hazard ratio (95% CI); *P* value
IRF Q1	Reference: no IRF or <Q2		
Q2	**2.32 (1.59–3.39); *P* < 0.001 [*P* < 0.001]**	**2.77 (1.94–3.96); *P* < 0.001 [*P* < 0.001]**	**2.71 (1.91–3.85); *P* < 0.001 [*P* < 0.001]**
Q3	**3.68 (2.53–5.36); *P* < 0.001 [*P* < 0.001]**	**3.81 (1.94–3.96); *P* < 0.001 [*P* < 0.001]**	**3.43 (2.44–4.82); *P* < 0.001 [*P* < 0.001]**
Q4	**3.59 (2.45–5.23); *P* < 0.001 [*P* < 0.001]**	**7.34 (3.83–14.1); *P* < 0.001 [*P* < 0.001]**	**3.64 (2.59–5.11); *P* < 0.001 [*P* < 0.001]**
*P* value linear trend	***P* < 0.001 [*P* < 0.001]**	***P* < 0.001 [*P* < 0.001]**	***P* < 0.001 [*P* < 0.001]**
SRF Q1	Reference: no SRF or <Q2		
Q2	1.17 (0.91–1.51); *P* = 0.230 [*P* = 0.271]	1.16 (0.90–1.51); *P* = 0.255 [*P* = 0.255]	0.91 (0.68–1.22); *P* = 0.550 [*P* = 0.550]
Q3	0.86 (0.65–1.13); *P* = 0.271 [*P* = 0.271]	**0.72 (0.54–0.95); *P* = 0.020 [*P* = 0.030]**	0.89 (0.68–1.19); *P* = 0.450 [*P* = 0.450]
Q4	**0.52 (0.38–0.71); *P* < 0.001 [*P* < 0.001]**	**0.59 (0.33–1.04); *P* = 0.001 [*P* = 0.002]**	0.74 (0.56–0.97); *P* = 0.031 [*P* = 0.081]
*P* value linear trend	***P* < 0.001 [*P* < 0.001]**	***P* < 0.001 [*P* = 0.003]**	*P* = 0.035 [*P* = 0.094]
PED Q1	Reference: no PED or <Q2		
Q2	1.12 (0.81–1.55); *P* = 0.484	1.14 (0.82–1.58); *P* = 0.430	1.51 (1.09–2.10); *P* = 0.014
Q3	1.38 (1.03–1.86); *P* = 0.030 [*P* = 0.091]	**1.49 (1.10–2.01); *P* = 0.010 [*P* = 0.029]**	**1.81 (1.31–2.49); *P* < 0.001 [*P* < 0.001]**
Q4	1.27 (0.95–1.69); *P* = 0.111 [*P* = 0.166]	1.37 (1.02–1.82); *P* = 0.034 [*P* = 0.052]	**1.72 (1.27–2.34); *P* < 0.001 [P < 0.001]**
*P* value linear trend	*P* = 0.054 [*P* = 0.094]	***P* = 0.012 [*P* = 0.037]**	***P* < 0.001 [*P* = 0.001]**

Hazard ratios for development of atrophy by Qs of different fluid volumes for each compartment with 95% CI (in round brackets); *P* values are presented as raw, and also as false discovery rate-adjusted values [in square brackets]; bold characters are used with false discovery rate-adjusted *P* values <0.05. Intraretinal fluid was associated with a significant risk of atrophy in higher volume Qs. Subretinal fluid showed a significant protective effect for atrophy development for higher volume Qs, especially in the central subfields. Pigment epithelial detachment was associated with a significantly higher risk of atrophy in the outer subfields, especially with higher fluid Qs. CI = confidence interval; IRF = intraretinal fluid; PED = pigment epithelial detachment; Q = quartile; SRF = subretinal fluid.

We found that higher Qs of IRF were associated with an increased risk of atrophy compared with Q1 (little or no fluid), with HR about 3.5 in all ETDRS rings (all *P* < 0.001).

The presence of SRF was associated with lower risk of atrophy, reaching Bonferroni-adjusted significance for Q4 (vs. Q1) at 1- and 3-mm ETDRS rings (HR, 0.5 and 0.6; *P* ≤ 0.001). Increasing PED volume was associated with the development of atrophy, with statistical significance in the 3-mm ETDRS ring for Q3 (*P* = 0.01) and in the 6-mm disc for Q3 and Q4 (both *P* < 0.001).

### Development of Fibrosis

These analyses included 763 eyes with no fibrosis at baseline, with 205 developing fibrosis during follow-up. The survival analysis estimated proportion free of fibrosis at 1 to 5 years was 85%, 77%, 67%, 57%, and 50%, respectively.

**Figure 3:**
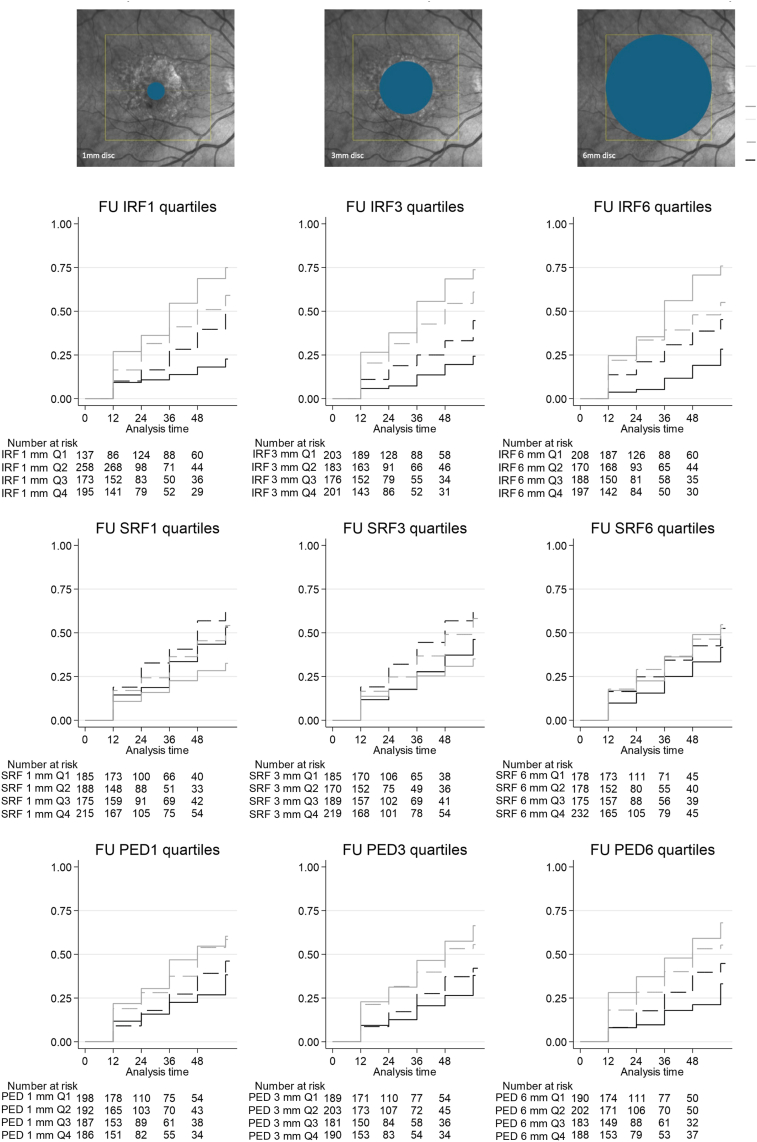
Risk of fibrosis along follow-up (months) for different fluid quartiles (Qs): intraretinal fluid (IRF; upper row), subretinal fluid (SRF; middle row), and pigment epithelial detachment (PED; bottom row) fluid volumes at 1, 3, and 6 mm (left, middle, and right columns, respectively). Line patterns are as follows: Q1, solid black; Q2, dashed black; Q3, dash gray; and Q4, solid gray. FU = follow-up.

Figure 3 and Table 3 show that fibrosis was also more likely in patients with more IRF at all locations (HRs, 2.28–5.12; Q3 and Q4 all *P* < 0.001). Subretinal fluid showed inconsistent associations across subfields, but did not reach statistical significance. The upper Qs of PED also increased the risk of fibrosis at all zones, with larger HRs in Q3 and Q4 between 1.83 and 3.19 than in Q1 (*P* ≤ 0.002).

**Table 3:** Fluid Volumes and Risk of Fibrosis

Fibrosis			
Volume Quartile	1 mm disc Hazard ratio (95% CI); *P* value	3 mm disc Hazard ratio (95% CI); *P* value	6 mm disc Hazard ratio (95% CI); *P* value
IRF Q1	Reference: no IRF or <Q2		
Q2	**2.28 (1.36–3.85); *P* = 0.002 [*P* = 0.003]**	**2.04 (1.27–3.28); *P* = 0.003 [*P* = 0.003]**	**2.42 (1.51–3.88); *P* = 0.001 [*P* = 0.001]**
Q3	**3.54 (2.10–5.98); *P* < 0.001 [*P* < 0.001]**	**3.51 (2.24–5.50); *P* < 0.001 [*P* < 0.001]**	**3.43 (2.22–5.42); *P* < 0.001 [*P* < 0.001]**
Q4	**5.12 (3.10–8.46); *P* < 0.001 [*P* < 0.001]**	**4.70 (3.06–7.21); *P* < 0.001 [*P* < 0.001]**	**4.87 (3.18–7.47); *P* < 0.001 [*P* < 0.001]**
*P* value linear trend	***P* < 0.001 [*P* < 0.001]**	***P* < 0.001 [*P* < 0.001]**	***P* < 0.001 [*P* < 0.001]**
SRF Q1	Reference: no SRF or <Q2		
Q2	1.39 (1.00–1.94); *P* = 0.051 [*P* = 0.076]	**1.68 (1.17–2.40); *P* = 0.005 [*P* = 0.014]**	1.46 (0.99–2.15); *P* = 0.055 [*P* = 0.063]
Q3	1.12 (0.79–1.57); *P* = 0.524 [*P* = 0.612]	1.41 (0.98–2.02); *P* = 0.063 [*P* = 0.095]	**1.61 (1.10–2.40); *P* = 0.013 [*P* = 0.040]**
Q4	0.64 (0.43–0.95); *P* = 0.028 [*P* = 0.076]	0.87 (0.58–1.31); *P* = 0.506 [*P* = 0.712]	**1.51 (1.04–2.17); *P* = 0.031 [*P* = 0.046]**
*P* value linear trend	*P* = 0.012 [*P* = 0.076]	*P* = 0.311 **[*P* = 0.018]**	***P* = 0.027 (*P* = 0.045)**
PED Q1	Reference: no PED or <Q2		
Q2	1.19 (0.79–1.79); *P* = 0.403 [*P* = 0.498]	1.23 (0.80–1.87); *P* = 0.347 [*P* = 0.412]	1.55 (0.99–2.41); *P* = 0.053 [*P* = 0.076]
Q3	**1.83 (1.26–2.67); *P* = 0.002 [*P* = 0.002]**	**2.08 (1.64–3.50); *P* < 0.001 [*P* < 0.001]**	**2.36 (1.55–3.59); *P* < 0.001 [*P* < 0.001]**
Q4	**2.03 (1.39–2.97); *P* < 0.001 [*P* < 0.001]**	**2.40 (1.64–3.50); *P* < 0.001 [*P* < 0.001]**	**3.19 (2.13–4.76); *P* < 0.001 [*P* < 0.001]**
*P* value linear trend	***P* < 0.001 [*P* = 0.001]**	***P* < 0.001 [*P* < 0.001]**	***P* < 0.001 [*P* < 0.001]**

Hazard ratios for the development of fibrosis by Qs of fluid volumes with 95% CI (in round brackets); *P* values are presented as raw, and also as false discovery rate—adjusted values [in square brackets]; bold characters are used with false discovery rate-adjusted *P* values <0.05. Higher quartiles of IRF volumes were associated with a significant risk of atrophy. Subretinal fluid showed a significant protective effect for fibrosis development for Q4 in the central subfield and a significant risk for fibrosis in the outer subfields progressively with higher SRF volumes. Pigment epithelial detachment volume was associated with a significantly higher risk of fibrosis in the outer subfields and with higher fluid Qs in all subfields. CI = confidence interval; IRF = intraretinal fluid; PED = pigment epithelial detachment; Q = quartile; SRF = subretinal fluid.

### Linear Trends of Fluid Behavior

The analyses of linear trends confirmed the progressive effect of fluid volume on both atrophy and fibrosis for IRF (all tests for trend *P* < 0.001); on the other hand, an inverse trend was observed for SRF regarding atrophy; in fact, compared with Q1, the upper Qs (Q3 and Q4) were associated with less risk of atrophy at both 1 and 3 mm (test for trend *P* < 0.001). A similar pattern was observed for fibrosis at 1 and 3 mm, but did not reach formal statistical significance (test for trend, *P* between 0.012 and 0.311). Regarding PED volume, positive trends were observed for atrophy, again reaching significance at 6 mm (*P* < 0.001); instead, increasing PED volume was significantly associated with fibrosis development at all locations (all *P* < 0.001).

### Predictive role of Baseline Values and Comparison with Repeated Biomarker Measures

Using Akaike information criteria and BIC, we found that modeling biomarkers with repeated measures yielded a better fit for all IRF and SRF models (lower Akaike information criteria and BIC by ≥10 points, favoring time-dependent models), both regarding atrophy and fibrosis as response variables, although not for all PED models.

Among nonfluid-related baseline covariates, age (HR, 1.33 per 10 years older; *P* = 0.001) and presence of SHRM (HR, 2.2; *P* < 0.001) reached the threshold for nominal significance for atrophy. Mixed/undefined MNV type was associated with a greater risk of atrophy than type 1, with borderline significance (HR, 1.59; *P* = 0.003). Age and SHRM did not predict the occurrence of fibrosis, whereas, compared with MNV type 1, we found that type 2 (HR, 1.63; *P* = 0.004) and mixed/undefined MNV (HR, 2.25; *P* < 0.001) increased fibrosis risk.

### Effect of Exposure to Fluid Volumes, Atrophy, and Fibrosis on VA

A subset of 552 eyes was analyzed to explore the effects of cumulative fluid volumes, presence of atrophy, and fibrosis on VA. Out of the original 1060 eyes recruited, 552 eyes displayed a minimum follow-up in VA of ≥12 months from baseline to be included in the analysis. Changes in VA are presented in Table 4. Lower baseline VA was associated with more VA improvement (all *P* < 0.001). Follow-up and its interaction with fibrosis were associated with worsening VA at all locations (all *P* < 0.001), whereas this was not observed with atrophy.

**Table 4:** Fluid Volumes, Atrophy, Fibrosis, and Risk of VA Decline

Covariate	1 mm	3 mm	6 mm
Baseline VA	**–0.1304**^‡^	**–0.1309**^‡^	**–0.1331**^‡^
Follow-up (per 1 mo)	**0.0077**^‡^	**0.0080**^‡^	**0.0084**^‡^
Atrophy time (per 1 mo)	0.0001	0	–0.0001
Fibrosis time (per 1 mo)	**0.0037**^‡^	**0.0036**^‡^	**0.0036**^‡^
IRF Q1	1 (ref)	1 (ref)	1 (ref)
Q2	–0.0085	0.0036	0.0037
Q3	**0.026^∗^**	**0.0418^∗^**	**0.0447^∗^**
Q4	**0.0904**^‡^	**0.0925**^‡^	**0.0908**^‡^
SRF Q1	1 (ref)	1 (ref)	1 (ref)
Q2	0.0048	–0.0052	–0.0052
Q3	**–0.0386^∗^**	0.0125	0.0282
Q4	**–0.0425^∗^**	–0.0063	0.0109
PED Q1	1 (ref)	1 (ref)	1 (ref)
Q2	**0.0455**^†^	**0.0449**^†^	**0.0591**^‡^
Q3	**0.0639**^†^	**0.0899**^‡^	**0.0707**^‡^
Q4	**0.0832**^‡^	**0.0923**^‡^	**0.0991**^‡^
Constant	**0.0583^∗^**	**–0.0993**^‡^	**–0.1129**^‡^

Change in VA according to exposure to the analyzed variables. Coefficients of VA are expressed in logarithm of the minimum angle of resolution. For each variable, notations of statistical significance are expressed after adjusting for false discovery rate (^∗^*P* < 0.05; ^†^*P* < 0.01; ^‡^*P* < 0.001). IRF = intraretinal fluid; PED = pigment epithelial detachment; Q = quartile; SRF = subretinal fluid; VA = visual acuity.

The highest Q of IRF (Q4) accounted for about +0.1 LogMAR of lower vision at all areas (all *P* < 0.001). Subretinal fluid volumes were associated with better vision of about –0.04 LogMAR only for Q3 and Q4 at 1 mm (*P* < 0.05). Higher PED volumes were always associated with reduced VA, worsening with higher Qs, and ranging from +0.04 to +0.9 LogMAR compared with Q1 (for Q2 at 1 mm, *P* < 0.01; for Q2 at 3 and 6 mm, and Q3 and Q4 at all locations, *P* < 0.001).

## Discussion

In this study, we provide a longitudinal analysis of OCT fluid volume biomarkers in the development of macular atrophy and fibrosis, supplemented with information on SHRM and MNV types. Based on a large series of treatment-naïve nAMD cases followed for a median of 2 years and up to 5 years, we found that IRF is a strong risk factor, whereas SRF may be a protective factor against atrophy, particularly in the central 1 mm of the macula. Although persistent and larger IRF volumes also increase the risk of fibrosis at all locations, SRF is associated with less fibrosis in the foveal center and more fibrosis in the 6-mm ETDRS circle, possibly indicating the increased chance of fibrosis in a large active lesion extending beyond the central 3 mm of the macula. However, these estimates did not reach our adjusted significance threshold.

Computation of fluid volumes using AI algorithms precisely monitors changes in fluid with comparable and higher reliability than human graders of different levels of expertise, making it possible to correlate precise quantitative values to other main outcome measures.^31^^,^^36^^,^^41^^,^^42^

Higher Qs of IRF in this model lead to a higher risk of both atrophy and fibrosis in all the ETDRS subfield areas. This finding is in accordance with previous literature, which highlights that the presence of IRF is strongly associated with deterioration in vision, leading to a lower chance of reversible visual recovery and a higher rate of atrophy and fibrosis.^43^^,^^44^

Although we found that higher Qs of SRF volumes in the 1- and 3-mm ETDRS subfields were associated with a lower chance of atrophy, there was a predisposition to the development of fibrosis in the 6-mm subfield. Furthermore, in our subanalysis on VA, the highest Q of SRF volumes was associated with less visual deterioration compared with the reference. These findings, which were specific to the central macula, are in accordance with previous literature, because SRF is considered less harmful than IRF and might also protect against atrophy and fibrosis in the foveal region.^43^^,^^45^^,^^46^ A small amount of SRF does not have adverse effects on VA, and outcomes have been shown to be comparable with those achieved when treatment has been carried out with the intent of complete SRF resolution, regardless of the MNV type.^25^^,^^47^, ^48^, ^49^ Nevertheless, SRF management, when present, should be addressed according to the clinical context. Recurrence of both SRF and IRF leads to nonreversible visual deterioration. When SRF is persistent, any new loss of vision carries a lower chance of recovery than a situation with no SRF. Furthermore, a higher volume of SRF was associated with VA loss, with findings indicating that treatment should be aimed at achieving a dry macula when possible.^29^ Moreover, greater fluctuations in IRF and SRF during the maintenance phase of anti-VEGF treatment have been associated with worse VA by 2 years.^24^^,^^30^ In summary, new occurrence of any fluid is likely to lead to vision loss, but small amounts of persistent SRF may be tolerated without compromising vision.^44^^,^^50^

The analyses of linear trends of HR across Qs of fluid confirmed the key role of IRF in the development of end-stage disease complications in nAMD. Regarding SRF, these analyses suggested that a thin layer of central fluid has a different meaning as a signal of MNV activity, compared with a larger pocket of SRF, which could, in fact, not predispose to direct development of atrophy and fibrosis in the fovea and subsequent visual deterioration. We also observed that higher PED volumes are a much stronger predictor of fibrosis than that of atrophy.

Regarding our findings relating to fluid localized in different regions of the ETDRS subfields, both IRF and PED have larger adverse effects on both atrophy and fibrosis when extending outside the central 3-mm ETDRS field; this may be representative of a larger MNV lesion size.

Longitudinal biomarker modeling in risk prediction is an interesting analytical option.^32^, ^33^, ^34^ Akaike information criteria and BIC modeling of IRF and SRF showed a good fit for the predictive effect of the volume of fluid on development of atrophy and fibrosis along follow-up, meaning that cumulative presence of IRF in the first phases of the disease contributes to atrophy and fibrosis and that SRF, especially in the central 1-mm, is associated with lower risk of development of atrophy and fibrosis. Therefore, we think that IRF can always be considered deleterious for the retina, and when present, will most likely increase the chance and speed toward atrophy and fibrosis. Subretinal fluid may intrinsically carry a protective effect because of the nature of the lesion. In type 1 MNV, which is generally linked to a more benign course, SRF is often the only manifestation and consequently has less deleterious anatomic effect on outer retinal architecture and organization. It has also been hypothesized that when SRF volumes are low and remain unchanged, they can have a protective effect on photoreceptors, delaying their degeneration and suggesting that there may be a balance between damaging and protective effects of the MNV and its exudation.^45^^,^^46^ When SRF is present in the outer rings of the ETDRS subfields, it may carry a higher risk of fibrosis because of the larger area of macula affected by the neovascular lesion.

In our study, higher Qs of PED volume were associated with a higher risk of atrophy and fibrosis, especially in outer retinal ETDRS subfields. This can possibly be explained by larger neovascular lesions having wider and larger PED, extending out of the 3-mm subfield, than lesions with no PED or smaller central lesions. Larger PEDs may predispose to local RPE atrophy and fibrosis on top of the PED, increasing the chance of atrophy and fibrosis because of the lesion itself or its collapse. When associated with markers of lesion activity, the probability of encountering atrophy and fibrosis increases and therefore accounts for a worse visual outcome.^24^^,^^51^ In addition, HRM in the sub-RPE space may also influence the development of fibrosis. This isolated effect should be investigated in further studies. Of interest, time-dependent modeling of PED volumes during follow-up yielded a similar fit to baseline PED values. A probable explanation is the relative stability of PED volumes in an eye, which we observed plotting longitudinal graphs of individual volumes. Given our findings, it may be worthwhile to try to decrease PED volumes over time to decrease the risk of the development of fibrosis and atrophy. One possible strategy may be to identify the smallest PED volume achieved (e.g., after loading dose) and maintain its stability without allowing further increase. Current treatment paradigms generally do not take this approach because only increases in IRF or SRF are used to trigger repeat treatment with “pro re nata” regimens or to reduce retreatment intervals if a “treat-and-extend” posology is being used.

Presence and quantity of SHRM were associated with greater volatility in fluid volumes, lower VA, and development of fibrosis, whereas type 2 MNV and macular hemorrhage were associated with the development of a fibrotic scar.^18^^,^^52^, ^53^, ^54^, ^55^, ^56^ In our analysis, SHRM at baseline was associated with an increased chance of atrophy, but not fibrosis. One possible explanation for this finding is that our analysis for this parameter is based on baseline features alone, and not on longitudinal values. Subretinal HRM may fluctuate in volume and reflect lesion activity and local exudation, which leads to outer retinal disruption, potentially followed by overlying photoreceptor atrophy in the long term. Alternatively, because type 1 membranes constituted the majority of this study sample, it is likely that any SHRM present resolved and therefore had little impact on the development of fibrosis.

At the same time, we found that type 2 and mixed/undefined MNVs were associated with the development of fibrosis, whereas only the mixed/undefined MNV type had a higher risk of atrophy. In this case, MNV typing alone cannot be an independent marker of prognosis because these qualitative data need to be correlated to other quantitative measures such as treatment regimen, adequacy of MNV activity restriction, and fluid volumes behavior in each compartment. In accordance with the literature, macular atrophy development is probably multifactorial, such as atrophy due to tissue disruption from the MNV itself, photoreceptor anomalies at baseline, chronic fluid exudation, and factors external to MNV itself, such as drusen and PED collapse.^11^^,^^20^^,^^54^^,^^55^

The longitudinal effect of fluid volumes, presence of atrophy, and fibrosis on VA was conducted on a subgroup of eyes with a follow-up of ≥12 months. We confirmed the adverse impact of IRF on VA and a partially protective effect of SRF on vision, especially in the 1-mm ETDRS subfield. Higher PED volumes were significantly associated with VA decline over time, highlighting the role of larger PEDs in the development of end-stage disease.

We also explored the time-dependent effect of atrophy and fibrosis on VA change over time and highlighted that fibrosis is the strongest factor associated with VA decline. This interaction was not found for atrophy, perhaps because of its slow progression.

As with all studies, our work has several strengths and weaknesses. A major strength is the use of time-dependent biomarker modeling in survival analysis to estimate HRs, with study eyes classified in 1 of 4 Qs of fluid volume at each follow-up point. This approach improved model fit compared with using only baseline values, except for PED volume, which undergoes little change during follow-up. Given the highly skewed distribution of fluid volumes, especially in the first 6 months of treatment, raw fluid volumes or their integral, as an AUC, used as a continuous measure at each time point, were not suitable for survival analysis in models predicting the onset of atrophy and fibrosis. The use of Qs to model the response variable means that relative effects across ordered categories at each time point were modeled as contrasts in fluid exposure, rather than making assumptions on the impact of linear or nonlinear functions of fluids, the distribution of which peaks at 0 and cannot be negative.

Moreover, for each eye, we used the integral (i.e., the AUC) of each fluid compartment during follow-up, assuming that the damage to the retina by pathologic fluid has a cumulative effect on the retinal tissues and their functioning. This is the first time that this approach has been used on fluid volumes, whereas it was recently used for VA data in assessing a consistent treatment response.^57^^,^^58^

We acknowledge that other options could have been used to model fluid effect, for example, adopting a lag that weighs more the impact of recent fluid, such as the last 3 or 6 months. We found that the use of lagged Qs yielded VA estimates that were similar to those obtained with standard Qs of AUCs of fluid. On the other hand, when continuous VA change was used as a response variable and continuous AUC and 3-month AUC of fluid were used as covariates, the lagged measure was inferior to the standard AUC. We therefore concluded that the predictive performance of the lagged measurements, which were restricted to the last 3 months of the index dates were not superior, and possibly inferior to the standard integral computation. This point may be justified by the nature of the disease itself. Although VA measures are associated with fluctuations depending on the presence of fluid, the overall longitudinal VA change over time can be better represented by cumulative fluid volumes contributing to the degeneration of the retinal layers and affecting the development of macular atrophy and fibrosis. The computation that took account of the full follow-up, rather than only the last 3 months, yielded a more robust estimate of the relationship. The comparison of different approaches to VA prediction is of little impact on the clinical perspective and is available upon request.

Nonetheless, in the absence of actual evidence, we considered the damage because of fluid as cumulative. We also acknowledge that the Q aggregation of the data at each monthly time point can only be adopted with large data sets with rather dense and regularly spaced time intervals.

Multiple imputation of missing data can be added as a sensitivity analysis in future studies.

Another weakness of this study is the lack of data pertaining to the frequency of intravitreal injections and the agent used because of the retrospective and automated data extraction from OCT machines.

Concerning data on VA, we were able to perform analyses only in a subset of patients with sufficient follow-up duration, which raises concerns about potential selection bias. We believe that these limitations are mitigated by the single-provider source of data, which may reduce the impact of confounding, both for anti-VEGF treatment delivery and VA acquisitions. A larger data set and longer follow-up for each patient are needed to strengthen these findings, together with a record of the number of injections.

Our results, based on anatomic and visual outcomes, provide further evidence that is in accord with a meta-analysis of vision outcomes, that is, that SRF is associated with better vision at the last follow-up, and that IRF is associated with worse vision.^48^

The present study confirms the effects of retinal fluid volumes on atrophy and fibrosis previously reported in the literature with stronger statistical evidence compared with previous studies, because it was possible to perform these analyses on a large volume of reliable data, quantified through AI algorithms, with a more precise approach, such as the use of time-dependent measures. With the introduction of new quantitative measures such as SHRM volume and with increasing precision of measurements, this approach may lead to a better comprehension of MNV phenomena and, subsequently, a reliable prediction of lesion prognosis.

The use of the ETDRS subfields to measure retinal fluid volumes is advantageous because it correctly accounts for the lesion size, morphology of the MNV, and provides a standardized assessment of general MNV.

Another important limitation of the present analysis is that SHRM, which may be composed of fibrin or entire blood fibrotic material, is not easily analyzed with current methods. In our data set, we graded fibrosis and atrophy as binary assessments performed by human graders based on the textural appearances of the images, rather than through automated analysis, which would have provided volumes. A longitudinal study with quantification of SHRM/fibrosis at every follow-up would better reflect the activity of the lesion. Because the current generation of algorithms does not reliably segment distorted atrophic and fibrotic retina in nAMD or provide distinction between these different pathologies, it was not possible to reliably capture the dynamic behavior of SHRM over time.

Fundus retinography and fluorescein angiography were not available, so fibrosis and atrophy were defined by SD-OCT characteristics alone, as well as the type of MNV. Although MNV assessment is easily achieved for common MNV types at baseline, it can be difficult for mixed MNVs and for advanced MNV lesions with disruption of retinal architecture. We did not directly measure the overall neovascular lesion size, but we based our analysis on ETDRS grid areas to estimate the extent of exudation and PED volume, which may partly compensate for the absence of this quantitative measure in our series.

## Conclusions

We report the results of a quantitative analysis of the changes in retinal fluid volumes over time, enabled by an AI segmentation system, in a large, routine clinical practice data set of eyes with nAMD treated with anti-VEGF therapy. Time-dependent survival analysis at multiple time points showed significant associations of SD-OCT-derived features with the development of atrophy and fibrosis. Demonstrating how certain SD-OCT biomarkers evolve during treatment, beyond the impact of pretreatment retinal morphology, may provide novel insight into disease mechanisms and aid the personalization of care. Cumulative IRF volume carries the highest risk for both macular atrophy and fibrosis development, followed by PED volume, while indicating a potential protective effect of SRF for atrophy development in the central macula. Cumulative higher IRF and the presence of fibrosis were associated with significant VA decline.
