# Supplementary material for: Fluid Volumes Longitudinal Modeling to Predict Atrophy and Fibrosis in Neovascular Age-Related Macular Degeneration
Source: Ophthalmol Sci. 2026 Apr 15;6(6):101190. doi: 10.1016/j.xops.2026.101190 (PMC13218248; doi:10.1016/j.xops.2026.101190)
Supplement: PDF Appendix S1 [file mmc1.pdf]

**SUPPLEMENTARY FILE 1**

**STATA CODE FOR MAIN ANALYSES**

**DATA AVAILABLE UPON REASONABLE REQUEST**

**Explanation of variables in the following text:**

- id: patient id in a long format, i.e. multiple records for the same eye over time, with time points as a column. Data are observational and not regularly spaced, but approximated to monthly interval (multiple measurement in the same interval must be merged)
- fu: consecutive months of follow-up for each eye, at which volume data and outcome(atrophy in this case) data are available
- irf1mm: AI-based values of intraretinal fluids within the central 1 mm at each available follow-up time, approximated to monthly intervals
- baseatrophy: eyes with atrophy at baseline will be excluded from this analysis
- atrophy: development of atrophy recorded as 0/1 at each time point available. It’s the failure event in survival analysis.

```
*****PREPARE INTEGRALS OF CENTRAL IRF FOR EACH SUBJECT
gen aucIRF1=.
    quietly  levelsof id, local(levels)
    foreach l of local levels {
           integ irf1mm fu if id== `l' , gen(x)
                replace aucIRF1=x if id== `l'
           drop x
     }
```

```
**PREPARE QUARTILES OF IRF BY FU STEP
gen q_irf1=.
   quietly   levelsof fu, local(levels)
    foreach l of local levels {
           egen x =cut(aucIRF1) if fu== `l' , group(4)
                replace q_irf1=x if fu== `l'
           drop x
      }

***LABEL    QUARTILES
lab def q 0 Q1 1 Q2 2 Q3 3 Q4
lab val     q_irf1   q


***KEEP EYES WITH NO ATROPHY AT BASELINE
drop if baseatrophy==1

 ****ST SET DATA WITH CLUSTER ON PATIENT ID
stset fu , fail(atrophy) id(id)

**HR FOR QUARTILES OF INTEGRAL IRF AT EACH FOLLOW-UP STEP
xi:stcox i.q_irf1 , robust

***test for time-dependence of quartile CATEGORIES
xi:stcox i.q_irf1 , robust tvc( _Iq_irf1_1 _Iq_irf1_2 _Iq_irf1_3 )

 **survival curves with tvc as an example (LITTLE DIFFERENCE with standard Cox model)
```

```
 scurve_tvc, generate(scurve0) at(_Iq_irf1_1 0 _Iq_irf1_2 0 _Iq_irf1_3 0)
tvc( _Iq_irf1_1 _Iq_irf1_2 _Iq_irf1_3)  texp(ln(_t))
  scurve_tvc, generate(scurve1) at(_Iq_irf1_1 1 _Iq_irf1_2 0 _Iq_irf1_3
0) tvc( _Iq_irf1_1 _Iq_irf1_2 _Iq_irf1_3)  texp(ln(_t))
 scurve_tvc, generate(scurve2) at(_Iq_irf1_1 0 _Iq_irf1_2 1 _Iq_irf1_3 0)
tvc( _Iq_irf1_1 _Iq_irf1_2 _Iq_irf1_3)  texp(ln(_t))
 scurve_tvc, generate(scurve3) at(_Iq_irf1_1 0 _Iq_irf1_2 0 _Iq_irf1_3 1)
tvc( _Iq_irf1_1 _Iq_irf1_2 _Iq_irf1_3)  texp(ln(_t))

 twoway line scurve0 scurve1 scurve2 scurve3 _tscurve, connect(J J J J)
ysca(rev) ylab(0 (.25) 1)


**LINEAR TREND OF QUARTILES
xi:stcox q_irf1 , robust

*EXTRACT P-VALUES FOR FDR
xi:stcox i.q_irf1 , robust
est store m1

*FDR adjusted p-values
etable, estimates(m1) cstat(_r_p, nformat(%12.10f))

**PLACE P-VALUES IN AN EXCEL FILE CALLED fdratrophy.xlsx
preserve
import excel fdratrophy , clear first case(lower)
 aefdr , bodysys(fam) event(num) pvalueadj(pvalues) fdrval(.05)
restore
```

```
**SURV CURVES
  sts graph, by(q_irf1)  fail xlab(0 12 24 36 48) risktab ti(FU IRF1
quartiles) saving( g1, replace)  legend(off)     plot1opts( lp(solid)
lcol(gs0) )    plot2opts( lp(dash)  lcol(gs0)) plot3opts( lp(dash)
lcol(gs10)) plot4opts( lp(solid)   lcol(gs10))

  **EXEMPLAR SENSITIVITY ANALYSIS ON 3-MONTH LAGGED  INTEGRALS
sort id fu
gen lagIRF1=.
    quietly  levelsof id, local(levels)
    foreach l of local levels {
                 replace lagIRF1= aucIRF1[_n]+ aucIRF1[_n-1]+ aucIRF1[_n-
2]+ aucIRF1[_n-3] if id== `l'
     }

** 3-MONTH LAGGED QUARTILES OF IRF 1  by FU STEP
gen lq_irf1=.
   quietly   levelsof fu, local(levels)
    foreach l of local levels {
           egen x =cut(lagIRF1) if fu== `l' , group(4)
                 replace lq_irf1=x if fu== `l'
     drop x
     }

*CORR between lagged and standard AUC
spearman lagIRF1 aucIRF1
tab  q_irf1  lq_irf1 , mis
preserve
drop if q_irf1==. |   lq_irf1 ==.
```

```
kappa  q_irf1  lq_irf1
restore

*COMPARE LAGGED AND STANDARD MEASURES
lowess auc lag
stset fu, fail(atrophy) id(id)
stcox i.q_irf1 , cluster(id )
stcox i.lq_irf1 , cluster(id)
```
