# Supplementary material for: Fluid Volumes Longitudinal Modeling to Predict Atrophy and Fibrosis in Neovascular Age-Related Macular Degeneration
Source: Ophthalmol Sci. 2026 Apr 15;6(6):101190. doi: 10.1016/j.xops.2026.101190 (PMC13218248; doi:10.1016/j.xops.2026.101190)
Supplement: PDF Figure S1 and Table S1 [file mmc2.pdf]

SUPPLEMENTARY MATERIAL – FILE 2

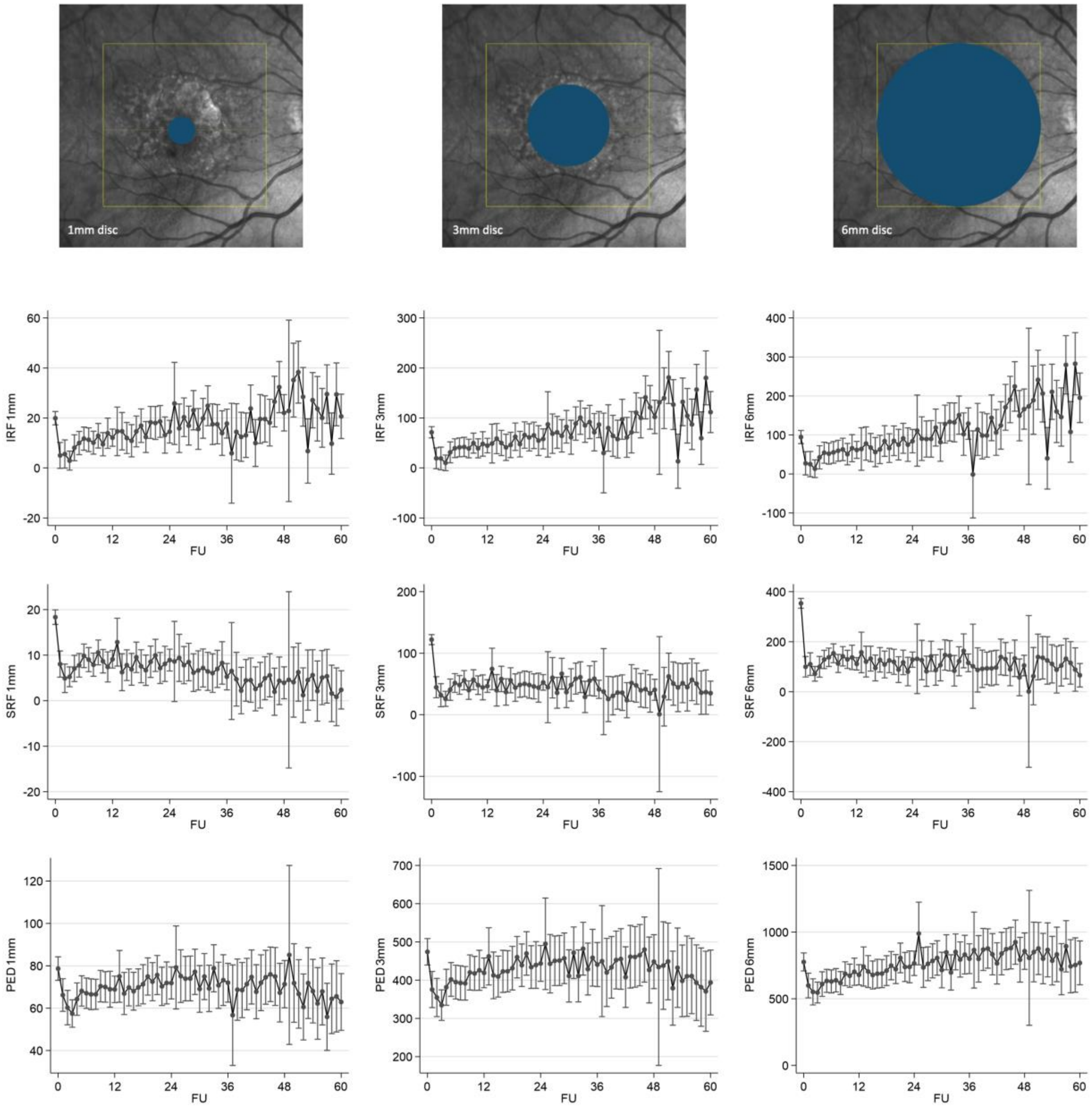

Supplementary Figure 1

Longitudinal presentation of different fluid compartments at each follow-up time, derived from linear mixed models.

Data on fluid volumes derived from AI-algorithms are displayed from baseline to 60-months follow-up. IRF at 1-mm, 3-mm and 6-mm ETDRS regions (top row left, center and right, respectively), after

an early decrease, showed an increase in trend along follow-up. SRF at 1-mm, 3-mm and 6-mm ETDRS regions (middle row left, center and right, respectively) showed a drop in the early phase, with trend for stability and slight decrease. PED at 1-mm, 3-mm and 6-mm ETDRS regions (bottom row left, center and right, respectively) showed an early decrease with relative stability along follow-up.

BASELINE FLUID QUARTILES CUTOFF VALUES					
	min value	Q1-Q2 cutoff	Median value	Q3-Q4 cutoff	max value
**Volume and subfield**					
**IRF 1mm**	0	0	0	3.9	599.5
**IRF 3mm**	0	0	0.41	23.49	3380.26
**IRF 6mm**	0	0	0.73	31.04	5781.44
**SRF 1mm**	0	0	0.15	4.04	302.80
**SRF 3mm**	0	0.48	4.68	41.50	1817.40
**SRF 6mm**	0	1.33	13.48	115.70	3399.69
**PED 1mm**	0	16.80	51.16	104.39	800.33
**PED 3mm**	0	112.98	307.49	617.41	5981.88
**PED 6mm**	0	149.93	430.54	1042.48	14980.15

Supplementary Table 1

Baseline fluid quartiles cutoff values

Baseline quartiles of fluid cutoffs are highlighted in the table, for intraretinal fluid (IRF) at 1,3 and 6-mm ETDRS regions (IRF 1mm, IRF 3mm, IRF 6mm, respectively), subretinal fluid (SRF) at 1,3 and 6-mm ETDRS regions (SRF 1mm, SRF 3mm, SRF 6mm, respectively) and pigment epithelial detachment (PED) at 1,3, and 6-mm ETDRS regions (PED 1mm, PED 3mm and PED 6mm, respectively).
