# Supplementary figures and images for: Fluid Volumes Longitudinal Modeling to Predict Atrophy and Fibrosis in Neovascular Age-Related Macular Degeneration
Source: Ophthalmol Sci. 2026 Apr 15;6(6):101190. doi: 10.1016/j.xops.2026.101190 (PMC13218248; doi:10.1016/j.xops.2026.101190)

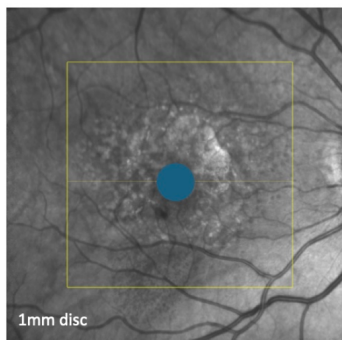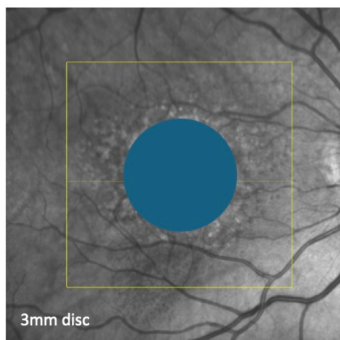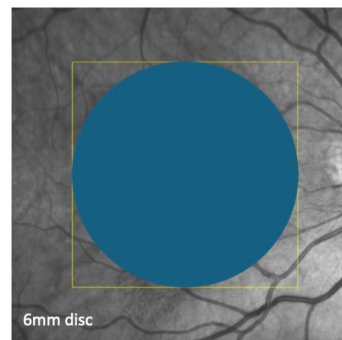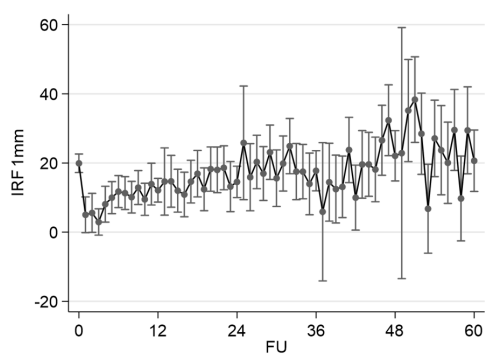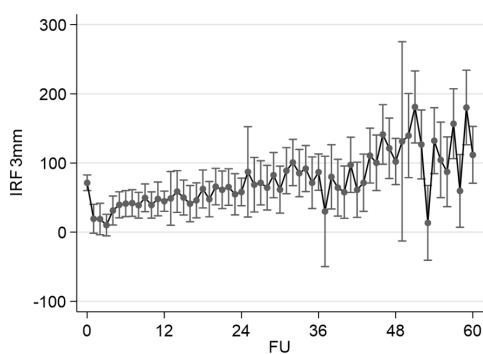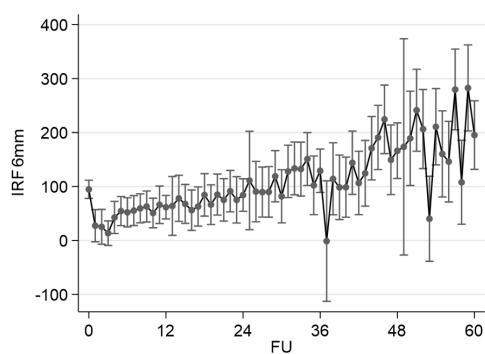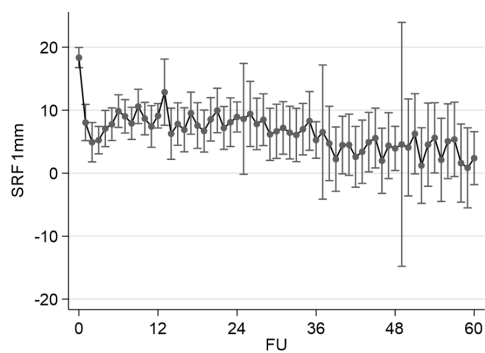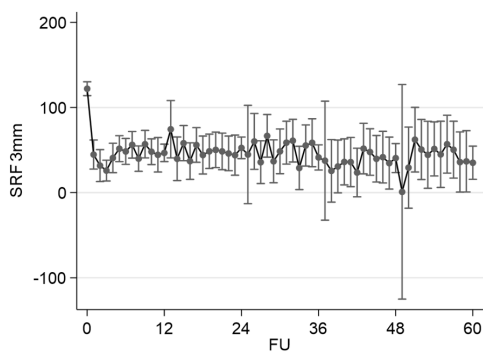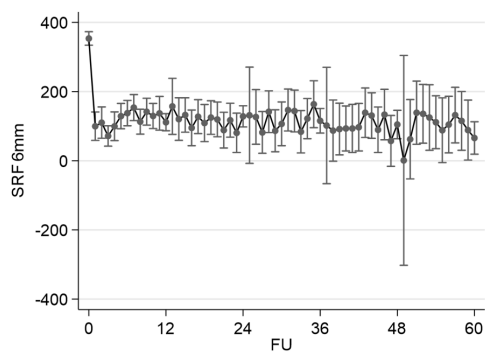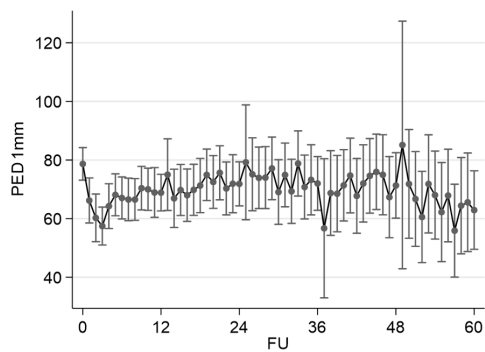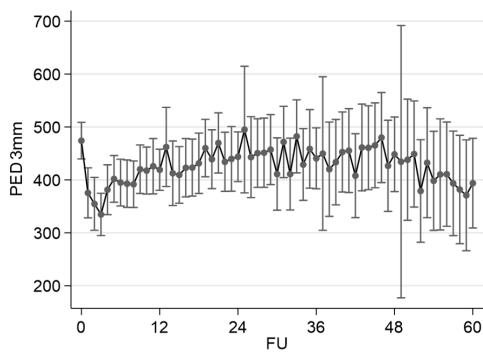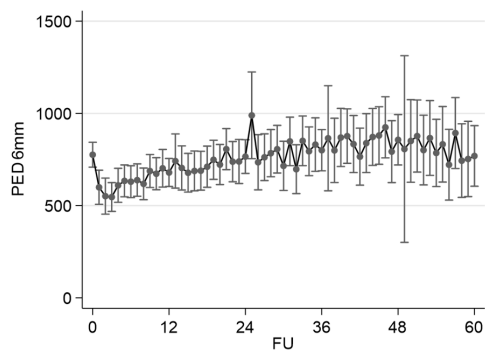

Supplement: PDF Figure S1 [file mmc3.pdf]
